# On the Use of the Forceps

**Published:** 1809-05

**Authors:** 


### *To the Editors of the Medical and Physical Journal.*

Gentlemen,

On the entrance of a young man into the practice of the medical profession, he is surrounded with difficulties; at a time when every little assistance is of consequence to him, he acts under the greatest disadvantages. Timidity, indecision, and youth, are all against him; and experience, that indispensable teacher of his art being wanting, he is obliged to trust alone to the too slender and inconsiderable knowledge which is afforded even by the most attentive reading and diligent attendance on public lectures.

If he is destined to follow his profession, as a general practitioner of medicine in the country, the scrutinizing publicity, inseparably attached to the inhabitants of a provincial town, increases the difficulties of his situation; every eye is upon him; and the poisoned tooth of detraction is always ready to fasten upon any seeming irregularity or failure in his practice.

At this period of his career, however, success in the different branches of his art is not of equal consequence to him. The unfortunate termination of a surgical or medical case, will in time be forgotten; but the *unlucky* death of a midwifery patient, (and chance has too great an influence in these cases) begets the greatest distrust, and often ruins his reputation and future prospects for ever.

In the progress of his education therefore, he will pay particular attention to the improvement of his knowledge in this department of medicine; and from the plain dogmatical matical directions given on the subject by books and teachers, he will often persuade himself, that he has acquired more knowledge than he really possesses. It is easy to say, and easy to understand, that in common cases, a plain and obvious practice is only necessary, as nature requires but little assistance; and that when certain difficulties arise, a certain line of conduct is to be instantly pursued. But how differently is a man situated, when reading in his study, or listening to a fluent lecturer, and when seated by the bed-side of his patient in labour. In the one case, his mind is calm and collected; in the other, it is ruffled by the cries of the woman and her friends, and agitated by anxiety for her safety, and for his own reputation. Hence, as a young practitioner, I cannot but be anxious, that every cause of embarrassment should, if possible, be removed out of his way.

When we consider the line of midwifery practice, in which a young man will probably be engaged, it will most certainly, except under particular circumstances, be found among the lower order of the people; but in addition to this, by far the greatest number of his patients will be young like himself, and consequently most of them will be pregnant of their first children. Now, it is perfectly well ascertained, that a woman's first labour, is, cæteris paribus, very different in its progress from any she may have afterwards. It would seem, as if Nature had implanted in the parts, through which the child's head passes, a certain rigidity and unsusceptibility of dilatation, which can only be overcome by the process of labour having once been gone through; and hence, that case in midwifery often arises, which, in the language of lectures, has acquired the name of a case of arrest; or, in other words, that case, in which the parts of the woman are in a dilatable state and covered with mucus, the head presenting properly and resting upon the perineum, but the powers of the woman's constitution, which were originally weak, almost totally exhausted by the length of the labour; producing quickness of the pulse, sighing, &c. with small, fatiguing, and unavailing pains.

This is a state, although of inconsiderable danger, yet one, in which danger will supervene if permitted to go on for above a certain length of time; therefore, it becomes the duty of the medical attendant, after that time, to free his patient from the irritating cause, by delivering her. For this purpose, the forceps is, by most experienced men, considered as the best instrument, and in books and lectures, its application is described as a very easy operation. Indeed, if it is lawful to judge by the practice, which teachers are in the habit of giving their pupils, on the machine, (and what are machines for?) it is of all things the easiest.

This, however, is very different indeed from what we find to be the case in actual practice. On the machine, if the right ear of the child can be felt by the finger of the operator, the introduction of the first blade of the forceps is perfectly easy, or even the second blade is far from being with difficulty introduced, although no direction can be taken by the other ear, in consequence of the frequent impossibility of feeling it. In the living body, the first blade is certainly applied in the proper situation with almost the same ease as on the machine, but not so the second. It not only frequently happens that the external opening of the vagina is not large enough to admit with ease the point of the second blade, but also, the folds of the vagina offer very considerable impediment to its passage. There is, however, another cause of difficulty in passing the second blade, which it is one great object of this letter to make observations upon.

The passage of the child's head through the pelvis is accurately and truly described by writers, and in most cases, where the labour is rapid at the time of delivery, the cone of integuments formed by the pressure, and which first presents to the finger, will undoubtedly be found over the saggittal suture near the posterior fontanelle, or somewhere on the occipital bone in the direction of the sagittal suture But in the protracted cases, as in those of arrest, this presenting cone of integuments will be found to have changed its place, and to be situated either on or about the prominent point of ossification of the parietal bone, or somewhere on the occiput in that direction, making the projecting part of the head to be passed over by the forceps, greater for one blade than the other; and as this greater projection falls to the lot of the second blade, it has in my practice very materially increased the difficulty of passing that blade, so much so, that in one case, it was found impossible to pass it while the first blade was in the vagina; and hence it was necessary to introduce the second blade before the first, which is certainly a great disadvantage, and much lessens the probability of the forceps being properly fixed on the head.

As this is an observation which I do not remember to have seen particularly mentioned, I have taken the liberty of of submitting it to your consideration, and also to the consideration of your correspondents, if you shall think it worthy of insertion in the Medical Journal, in the hopes that either you or they may, by more extensive experience, appreciate its truth, and if necessary, offer what remarks may be thought right, for the prevention of the inconvenience.

For my own part, I can at present think of nothing but desiring the woman to lie for several of the last hours of the labour upon her right side, as it seems probable that the sinking down of the left side of the child's head depends upon the woman having lain during the labour upon her left side, that being the most convenient situation for the practitioner to take his examinations.

I am, &c. H.

*Feb.* 9, 1809.

